# Comparative study of elastic properties measurement techniques during plastic deformation of aluminum, magnesium, and titanium alloys: application to springback simulation

**DOI:** 10.1007/s11012-024-01918-8

**Published:** 2024-11-29

**Authors:** J. A. Nietsch, A. C. Ott, G. Watzl, A. Cerny, F. J. Grabner, C. Grünsteidl, J. A. Österreicher

**Affiliations:** 1 LKR Light Metals Technologies, Austrian Institute of Technology, Lamprechtshausenerstr. 61, 5282 Ranshofen, Austria; 2 Research Center for Non-Destructive Testing GmbH, Altenberger Straße 69, 4040 Linz, Austria

**Keywords:** Young’s modulus, Springback, Forming, Mechanical properties, Light metals

## Abstract

Reliable determination of the elastic moduli of metals can be quite demanding, especially as the apparent elastic modulus of metals is known to decrease with deformation. Traditionally, this dependence on plastic strain has been investigated through various tensile tests, but discrepancies persist across the different approaches. Here we compare several tensile test-based evaluation protocols based on loading-unloading experiments to measure the change in elastic moduli of the light metal alloys AZ31B, EN AW-6082, and Ti–6Al–4V during tensile deformation. Additionally, the initial Young’s modulus determination via tensile testing, three-point-bending experiments, contact-free laser ultrasonic zero-group-velocity plate resonance, and piezoelectric contact ultrasonic time-of-flight measurements were compared. The results reveal non-negligible differences in the strain-dependency of elastic moduli between the determination techniques. Additionally, the laser ultrasound measurements demonstrate an improved accuracy and repeatability for the determination of the initial elastic moduli of light metal sheets. The benefit of considering the reduction of the elastic moduli in finite element springback simulation of three-point-bending tests is demonstrated and the use of the chord modulus is found to be generally most appropriate.

## Introduction

The knowledge of elastic parameters plays a major role in estimating the springback behavior in sheet metal forming and bending processes. In particular, the values used for the Young’s modulus influences the results of numerical forming simulations [1]. Often, finite element (FE) simulations still use constant values for the modulus of elasticity, which are commonly taken from the general literature, ignoring the weakening of the elastic modulus caused by plastic deformation.

Neglecting this effect may lead to increased differences between simulation results and reality: Cleveland and Ghosh [2] showed for the aluminum alloy EN AW-6022-T4 and a high strength steel that a 10–20 % error in the springback estimation can occur. Conversely, incorporating the phenomenon of the elastic moduli reduction during plastic deformation, which is based on an anelastic material behavior, can increase the accuracy of finite element analyses [3–9].

It is uniformly understood that the anelasticity of metals at low temperature is related to the motion of dislocations [10]. The reduction of the elastic modulus is mainly caused by micro-plastic deformations [11]. During the plastic deformation, free dislocations move along the slip planes until they are barred, which causes them to pile up. These dislocation pile-ups can dissipate when the stress is removed, leading to a small portion of elastic strain. This arises from mobile dislocations which are able to move in response to the internal repulsive forces between them, as soon as the external force is removed [12]. A reasonable way to describe this was developed by Cleveland and Gosh [2], who divided the unloading behavior in several stages. First, the stress level drops to the internal strength of the material, which is equals the back-stress of pile-ups plus the barrier strength. Then, the piled up dislocations retreat, before additional dislocations, such as dislocations who are intertwined in the cell walls [2] or from dislocation tangles [12], become loose and start to move in the reverse direction. Thus, a greater strain release can be expected in a third stage, leading to an exponentially increasing compliance [2]. Based on the characterization efforts of other researchers [13, 14], it was concluded that the observed reduction of the elastic modulus in unloading is mainly attributed to micro-plastic strain caused by displacement of mobile dislocations. Since the dislocation density tends to increase with an increase in deformation, the contribution of the dislocation behavior to the non-linear springback also increases with increasing forming strain [12]. Nevertheless, the increase of the micro-plastic strain depends on the material and a simple correlation with the strain is not possible [15].

Therefore, the hardening model used in FE simulations plays an important role in predicting the magnitude of the springback. Eggertsen and Mattiasson [16] validated the applicability of several hardening laws using the NUMISHEET’93 benchmark problem for the springback predictions of several steels. Their results not only revealed the necessity to use a hardening model that can properly describe the Bauschinger effect, the transient behavior, and the permanent softening effect, they also demonstrated the need to incorporate the effect of elastic degradation with increasing plastic strain.

Consequently, precise measurements of the elastic moduli and their changes are desirable. There are several determination techniques available, which are either based on mechanical testing or indirect techniques such as measuring the speed of sound [17–19] or plate resonance frequencies [20]. Determining the superior arrangement for a Young’s modulus test presents a challenge due to the absence of a physically grounded justification for selecting the optimal method [21].

However, several methods are relatively reliable for the determination of the initial Young’s modulus, while the detection of deformation-induced changes in elastic moduli via indirect techniques can be very challenging. In this work, we refer to the initial elastic modulus as Young’s modulus, while moduli measured after plastic deformation will be referred to more generally as elastic moduli. Techniques based on the generation and detection of plate (Lamb) waves, for example, typically assume isotropic material behavior [22, 23] and would therefore require special mathematical modeling, when applied to strained materials [24]. Therefore, mechanical tests are commonly used for elastic moduli determination within the plastic deformation regime. Several experimental procedures based on bending, torsion and tensile tests have been developed and are continuously modified with the aim to improve their accuracy. Combinations of different loading modes, such as torsion and tension [25], cyclic torsion tests [26], inverse modeling techniques, such as coupling of FE simulations and Matlab®optimization algorithms of bending experiments [27], and several methods for calculating strain dependent values from multiple unloading and reloading cycles of specially adapted tensile experiments [9, 15, 17] were applied. These methods exhibit major differences in the experimental set up and the processing of the measured force and displacement curves, thus affecting the material characterization results.

One way of determining the apparent elastic modulus during plastic deformation would be a simple linear regression in unloading experiments [28]. However, such a regression is not able to sufficient describe the complex nonlinear unloading behavior of metals after plastic deformation, which consequently motivated the development of several alternative approaches. Kim et al. [15] determined two moduli, named reloading and unloading modulus, from each cycle of the true stress-true strain curves obtained from a uniaxial tensile test. Thereby, their unloading modulus corresponds to the chord modulus of other researchers. Abvabi et al. [9] calculated three moduli, named chord, unloading, and reloading modulus, on true stress/strain curves using slightly different regression boundaries for analyzing the reloading modulus. Instead of neglecting stresses below 25 MPa like Kim et al. did, Abvabi et al. used a 50 MPa cutoff as the lower and upper boundary in order to remove the influence of nonlinearities on the moduli calculation. To obtain a more accurate description of the unloading and reloading behavior of metals, Chen et al. [17] calculated five moduli from the true stress-true strain curves. Yoshida et al. [29], on the other hand, omitted the determination of the reloading modulus and used four unloading moduli from the true stress/strain curves under in-plane cyclic tension-compression loads instead. All these methods differ in the treatment of early-stage effects that arise from the transition from unloading to reloading, as well as in the choice of data points used to determine the moduli via linear regression. The methods of [9, 17, 29] and their implementation in the herein conducted experiments will be discussed in more detail in the following section.

These determined elastic moduli are key values that describe the material behavior, which consists of an elastic and a non-elastic part, and are calculated according to Chapter 2. The value and the amount of non-elastic strain contained in the modulus of each key value depends on its calculation, for example it is considered that the chord modulus effectively considers the whole non-elastic part of the deformation [15]. Nevertheless, with increasing strain, a decrease of these key values to a saturation level can be witnessed for several materials, which includes steels [9, 15, 17, 26, 28–32], aluminum [2, 3, 33, 34], titanium [4, 31, 35–37], and magnesium alloys [25, 38]. Further investigations also revealed that the saturation level of sheet metal is influenced by the sampling direction [11, 12]. For example, the results of Liu et al. [4] show that the saturation level of 2 mm Ti–6Al–4V sheet is higher in rolling direction than in transverse and diagonal direction, which also corresponds to the witnessed differences in the initial Young’s moduli caused by the sampling direction (i.e., the material is anisotropic). In contrast to these results, Khayatzadeh et al. [35] reported for sheets made of commercially pure titanium the opposite, as the measured elastic modulus in rolling direction was continuously lower than in the transversal and 45$^\circ$ direction.

Another influence is the forming temperature; as the temperature increases, the reduction of the elastic moduli caused by increasing strain decreases. This can be expressed in the ratio of the initial Young’s modulus to the saturated moduli of elasticity or simply the maximum achievable reduction of these moduli. Ma et al. [37] described a drop of the maximum chord modulus reduction from 18 % at room temperature to 6 % at 400 $^\circ$C for the titanium alloy Ti–3Al–2.5V. This weakening of the nonlinear behavior of the strain-related chord modulus degradation could be caused by changes in the dislocation behavior affecting the temperature-dependent evolution of the reversible dislocation density and the mean free path of the dislocations. A comparison of this drop for different materials reveals that the amount of the moduli reduction varies with different materials [12, 15, 37, 39] and is also influenced by the microstructure [12, 40]. Depending on the microstructure of a material, the drop of the apparent elastic modulus and the strain needed to reach the saturated regime of the elastic moduli is able to show major differences. Such differences can be caused by variations of the martensite volume fraction and the martensite hardness [40]. Some investigations additionally brought to light that the reduction of the elastic moduli, caused by plastic deformation, depends on the loading direction or rather the type of loading [1, 31], while the strain rate seems to have no effect [15].

So far, it is unclear which of above-described procedures is more suitable in describing the strain-dependent behavior of the elastic modulus of light metal alloys, as most of them where initially developed for steel. At the same time, the application of these established procedures can lead to differences in the determined elastic properties, thus creating uncertainties in the FE based springback estimation. Depending on the material and the used moduli, such as unloading or chord modulus, the discrepancies between experiment and simulation can lead to a higher or lower springback prediction [9].

In summary, the modeling of the springback behavior is very complex and can be influenced by several parameters. In addition to the characteristic material properties, which include the Young’s modulus, the hardening and recovery behavior, and process parameters, such as the blank holding force [41], play an important role. To make matters more difficult, the time of the practical test evaluation must also be taken into account for verifying the validity of the models, due to the often observed time dependency of the springback [1, 34, 42]. As a consequence, this work aims to reduce some uncertainties in the springback prediction of light metals via the comparison of several tensile test-based determination techniques for the estimation of the deformation-dependent elastic moduli.

## Theory and methods for determining deformation-dependent elastic moduli

In this study, four different methods were used to determine elastic moduli directly from tensile tests, including three described in previous literature [9, 17, 29], and a method newly developed by the authors. This new method is based on a slightly modified hysteresis loop of the international standard for tensile testing EN ISO 6892-1 and a quadratic regression method to determine the relevant points of the hysteresis loop (i.e., intersection points). In contrast to the ISO standard, this method uses true stress and true strain for calculating the different elastic moduli in order to be more comparable with the other implemented methods, which were taken from the scientific literature.

**Fig. 1:**
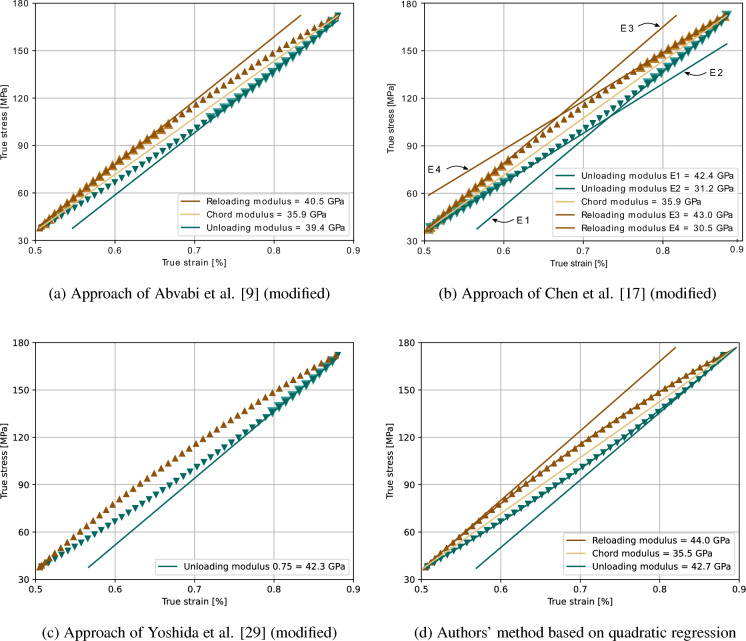
Illustration of the four methods used to determine the strain-dependent elastic moduli. (Color figure online)

In all methods, hysteresis loops are generated by applying and releasing tension to the specimen in an alternating manner (Fig. 1). The unloading curve (green triangles pointing downward) includes all data points that occur during the reduction of tensile stress, while the reloading curve (brown triangles pointing upward) results from the data points that occur after reloading, not exceeding the associated unloading curve (first point of unloading curve).

Three moduli can be determined for each loop. The chord modulus is defined as the gradient of the straight line between the two starting points of the curves and the unloading and reloading moduli are calculated by linear regression of the unloading and reloading curves, respectively. It should be noted that the evaluation can be carried out in the initial or final range of the curve (Fig. 1b). To avoid measurement errors, a predefined directional load change at a tensile load of 1000 N during the unloading step was applied. This lowest unloading stress corresponds to a stress level of approximately 25 MPa, which is less than 15 % of the yield strength of the investigated materials before unloading.

A short description of the tensile-based elastic moduli determination methods can be taken from Table 1 and Fig. 1a–d.

**Table 1:** Overview of employed methods

Method	Calculated moduli	Remarks
Abvabi et al. [9] modified	Chord, unloading, reloading	Differences in lowest unloading stress
Chen et al. [17] modified	Chord, 2x unloading, 2x reloading	Differences in lowest unloading stress
Yoshida et al. [29] modified	3x unloading	Conducted without compression stress
Authors’ method	Chord, unloading, reloading	Quadratic regression at the chord ends

Therein, true stress and true strain were always used to determine the corresponding moduli of elasticity. The relationship between true stress/true strain and the engineering stress/engineering strain, which is specified by several tensile test specifications, e.g. EN ISO 6892-1 [43], is defined as:

1 $$\begin{aligned} \epsilon _{\text {true}} = \ln (1 + \epsilon _{\text {eng}}) \end{aligned}$$

and

2 $$\begin{aligned} \sigma _{\text {true}} = \sigma _{\text {eng}} (1 + \epsilon _{\text {eng}}) \end{aligned}$$

At low plastic strains, differences in the true and engineering stress and strain values are negligible. However, due to the fact that the true stress considers the change in the sample cross section during deformation and an increasing difference between engineering and true strain at higher plastic strains, significant differences between the methods may arise at higher strains.

To illustrate the methods in detail, the first hysteresis loop of an AZ31B tensile test is evaluated as an example and discussed below. Figure 1a shows the procedure according to the cutoff technique by Abvabi et al. [9] for calculating the unloading modulus, which uses the data points of the unloading curve from the upper point to a stress value of $\sigma :=(\sigma _1-\sigma _2)/2$ (highlighted in Fig. 1a) for linear regression. Here, $\sigma _1$ denotes the highest stress value and $\sigma _2$ the lowest stress value in the hysteresis loop. Similarly, the initial data points of the reloading curve up to a maximum value $\sigma$ are used for the reloading modulus. To take an offset (by changing the stress direction) into account, a threshold value was predefined in the initial range of the unloading- and reloading curves. Abvabi et al. [9] define 50 MPa for steel and the same value is used for Ti–6Al–4V, whereas 25 MPa is more suitable for EN AW-6082, and 15 MPa for AZ31B. Finally, the chord modulus is determined by using the upper and lower point of the loop (same points providing $\sigma _1$ and $\sigma _2$).

Similar to Abvabi et al. [9], the modified Chen et al. [17] approach (Fig. 1b) uses datasets for linear regression specified by $\sigma _1$ and $\sigma _2$. The unloading modulus E1 includes unloading data points greater than $2(\sigma _1-\sigma _2)/3$, while the unloading modulus E2 includes those less than $(\sigma _1-\sigma _2)/4$. Consistently, the reloading modulus E3 results from reloading data up to a stress value of $(\sigma _1-\sigma _2)/3$ and the reloading modulus E4 from those greater than $3(\sigma _1-\sigma _2)/4$. The authors do not use a threshold value for the offset, as the procedure by Chen et al. [17] is not applicable here. This is due to the different offset properties and the fact that each loop is not completely unloaded. The chord modulus is calculated via the endpoints of the loop as before.

In the implemented approach based on Yoshida et al. [29], several unloading moduli are required. To obtain these values, the largest tensile stress value $\sigma _\text {max}$ before decreasing stress is determined (Fig. 1c). An offset is removed by applying a threshold of $0.95\ \sigma _\text {max}$, and subsequently, unloading moduli are calculated by performing linear regression on the unloading data greater than $0.25\ \sigma _\text {max}$, $0.5\ \sigma _\text {max}$, and $0.75\ \sigma _\text {max}$. Due to the fact that the stress level during the unloading never reached 0 MPa, only three of the four moduli used by Yoshida et al. were determined.

In order to validate the transferability of the international tensile test standard [43] for the determination of the strain dependent Young’s modulus, a new approach was developed. This alternative approach for determining elastic moduli is shown in Fig. 1d. Thereby, the unloading and reloading curves are fitted using a second-degree polynomial. The reloading modulus is determined as the gradient of the reloading function at the lower intersection of the two polynomials, while the unloading modulus is determined as the gradient of the unloading function at the upper intersection. To obtain the chord modulus, the straight line passing through the two intersection points of the polynomials is used.

## Experimental

We employed sheets of three commonly used light metal alloys to characterize deformation induced changes of the elastic material behavior: Al-Mg-Si alloy EN AW-6082 in the peak-aged temper (T6), Mg-Al-Zn alloy AZ31B, and Ti–6Al–4V (a.k.a. Ti64 or Ti Grade 5). To ensure comparability, the thickness of all investigated sheets was close to 2 mm (see Table 2).

**Table 2:** Properties of the investigated sheet materials

Material	Sheet thickness [mm]	Yield strength [MPa]	Ultimate tensile strength [MPa]	Density [kg/${\hbox {m}}^{3}$]
EN AW-6082-T6	1.98±0.02	337.9±3.0	366.2±4.4	2,701.30±11.30
AZ31B	2.00±0.02	166.2±2.3	257.4±2.0	1,774.35± 9.10
Ti–6Al–4V	2.10±0.02	972.3±2.1	1,028.9±2.0	4,441.40±12.00

The Young’s moduli of these sheets were investigated via piezoelectric contact ultrasonic time-of-flight measurements (US-tof) and laser ultrasonic zero-group-velocity (ZGV) [44] plate resonance measurements (LUS-res) in a load-free condition. Additionally, the initial Young’s modulus was determined from bending and tensile tests, while the deformation-dependent elastic modulus was only determined from tensile experiments.

All tensile and bending test specimens were cut in rolling direction and tested on universal testing machines from Zwick/Roell with a maximum applicable force of 100 kN or 250 kN (Z100 and Z250 machines, respectively). Both testing machines were equipped with sensor arm extensometers from Zwick/Roell (type 066550 and makroXtens HP, respectively), which enable the precise elongation measurement during tensile and cyclic testing up to a maximum measuring range of 450 mm. The knife edges were attached automatically and fixed in their initial position using a clamping force.

For the uniaxial tensile tests, standardized samples type DIN 50125 H20$\times$80 with a gauge length of 80 mm were used. The LUS-res measurement of the initial Young’s modulus were conducted on 70 $\times$ 70 mm sheets, while the US-tof measurements were conducted on 25 $\times$ 75 mm sheet metal strips.

### Determination of the initial Young’s modulus

The determination of the initial Young’s modulus was carried out via ultrasonic based time-of-flight (US-tof) measurements, laser ultrasonic measurements, tensile tests and three point bending experiments. Both ultrasonic measurements are indirect determination techniques, which are based on the known empiric correlations between the elastic constants Young’s modulus *E*, shear modulus *G*, Poisson’s ratio $\nu$, the density of the material and sound velocities [18–20]. In general, the sound velocities are related to the elastic moduli via the density as follows:

3 $$\begin{aligned} E= & 2 G(1+\nu ) \end{aligned}$$

4 $$\begin{aligned} G= & \rho c_{\text {trans}}^2 \end{aligned}$$

Furthermore, the longitudinal and shear-wave velocities are linked via the Poisson’s ratio, see Eq. (3)

5 $$\begin{aligned} c_{\text {trans}}=c_{\text {long}}\sqrt{\frac{1-2 \nu }{2(1-\nu )}} \end{aligned}$$

At first, the nondestructive US-tof measurements were conducted with a portable phased array ultrasonic flaw detector type Phasor XS from GE using a longitudinal ultrasonic transducer type CLF-4 (15 MHz) and transversal ultrasonic transducer type K7KY (7 MHz). Based on the manual sound velocity measurements, the equation given by McIntire et al. [45] was used to calculate the Young’s modulus.

6 $$\begin{aligned} E=\rho c_{\text {trans}}^2\frac{3 c_{\text {long}}^2 -4 c_{\text {trans}}^2}{c_{\text {long}}^2 - c_{\text {trans}}^2} \end{aligned}$$

In order to further consider the effect of different determination methods, laser ultrasonic (LUS) measurements were conducted. A Bright Solutions Wedge HB-1064 laser with a wavelength of 1064 nm was used for the thermoelastic generation of the sound impulses (elastic waves) in the samples. The displacement of the surface due to these elastic waves was detected by a laser vibrometer (Sound & Bright Tempo) at the same point and recorded by an oscilloscope. This arrangement enables the generation and detection of at least 2 ZGV resonances in the samples. From the ratio of these resonance frequencies ($f_\text {ZGV1}$, $f_\text {ZGV2}$), $\nu$ can be determined directly, assuming isotropic material properties [20, 22]. In order to utilize the detected ZGV resonances for the determination of the Young’s modulus, the relation between the longitudinal sound velocity, the material thickness *s*, and $f_\text {L1}$, which is is the first longitudinal thickness resonance frequency, must also be taken into account. Although we do not measure the fundamental longitudinal thickness resonance frequency $f_\text {L1}$ directly, it can be calculated from the lowest order ZGV mode frequency $f_\text {ZGV1}$ and $\nu$ with

7 $$\begin{aligned} f_\text {L1} = f_{\text {ZGV1}} / \beta (\nu ) \end{aligned}$$

using the shape factor $\beta (\nu )$ [46], which only depends on $\nu$ and can be deduced from numerical solutions to the Rayleigh-Lamb equations [22]. Therefore, $c_\text {long}$ can then be determined using:

8 $$\begin{aligned} c_\text {long}=2 s{f_{\text {L1}}} \end{aligned}$$

In addition to the measured ZGV resonance frequencies, the sheet thicknesses and densities displayed in Table 2 were used for the final calculation of *E* and *G* (Eqs. 3–5). These values were measured during this investigation via an external micrometer, tensile tests, and the Archimedes principle. Consequently, Table 2 contains averaged values and the corresponding standard deviation. For a more detailed explanation of the method and signal processing techniques we refer to previous work [23].

The tensile test used for the mechanical determination of the initial Young’s Modulus were conducted according to EN ISO 6892-1 [43]. Consequently, the Young’s Modulus was calculated via linear regression of the stress and strain data within the lower elasticity regime. Only data points between 10 % and 40 % of the yield strength were considered for the regression. Next to this classical approach, a the Suttner-Merklein technique for determining the Young’s modulus [47], which aims to increase the accuracy of estimation of elastic properties in tensile testing by utilizing the deformation work, was also used.

**Fig. 2:**
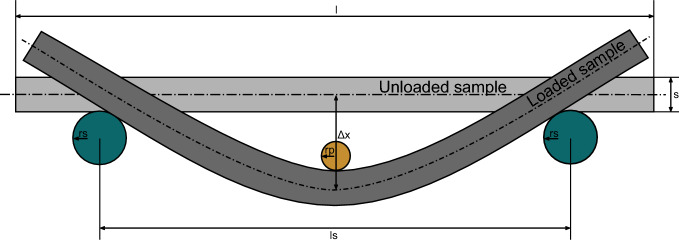
Illustration of the three-point bending experiments

Additionally, bending experiments (see Fig. 2) were conducted on the Zwick Z100 using a punch radius $r_\text {p}$ of 1.5 mm and a support radius $r_\text {s}$ of 2.5 mm. The samples used had a length *l* of 175 mm, a width *w* of 20 mm and a thickness *s* according to Table 2. The samples were placed on the two steel supports and deformed with a constant speed of 1 mm/s. Taking into account the span length $l_\text {s}$ of 80 mm and the method described by Sun et al. [48], the Young’s Modulus of the elastic regime was calculated.

### Determination of elastic properties during continuous plastic deformation

Standardized tensile tests were used for flow curve determination and a rough estimation of possible loading-unloading-loading cycles prior to the conduction of the adapted tensile experiments, which was necessary to ensure that all loading cycles were conducted in the area of uniform sample elongation. The targeted plastic unloading strains were 0.5 %, 2.0 %, 3.5 %, 5.0 % and 7.5 %. However, due to the low uniform elongation of the titanium alloy, the tensile experiments for Ti–6Al–4V were conducted until a maximum plastic strain of 6.3 % was reached. A comparison of several tensile test-based Young’s modulus determination methods was conducted. For further information regarding the applied methods and a description of the corresponding methods, see Chapter 2. At least five loading-unloading tensile experiments per material were conducted.

### Application of the determined values in springback simulation of a bending process

In order to validate the applicability of the herein determined elastic moduli, the FE analysis method was used to simulate the springback after three-point bending experiments for EN AW-6082-T6, AZ31B, and Ti–6Al–4. For experimental validation, these three-point bending tests using a geometry of 80 $\times$ 20 $\times$ 2 mm were conducted on a Zwick Z100 tensile testing machine similar to VDA 238-100 [49]. Thereby, the roller distance (i.e., the span length l$_s$) was 34 mm, the roller diameter was 30 mm and the punch radius r$_p$ was 0.4 mm. A punch velocity of 20 mm/min was used and the test was stopped after a displacement of 4.5 mm. A photograph was taken under load and immediately after unloading using a tripod-mounted smartphone camera. The bending angles were determined using ImageJ 1.54f.

**Fig. 3:**
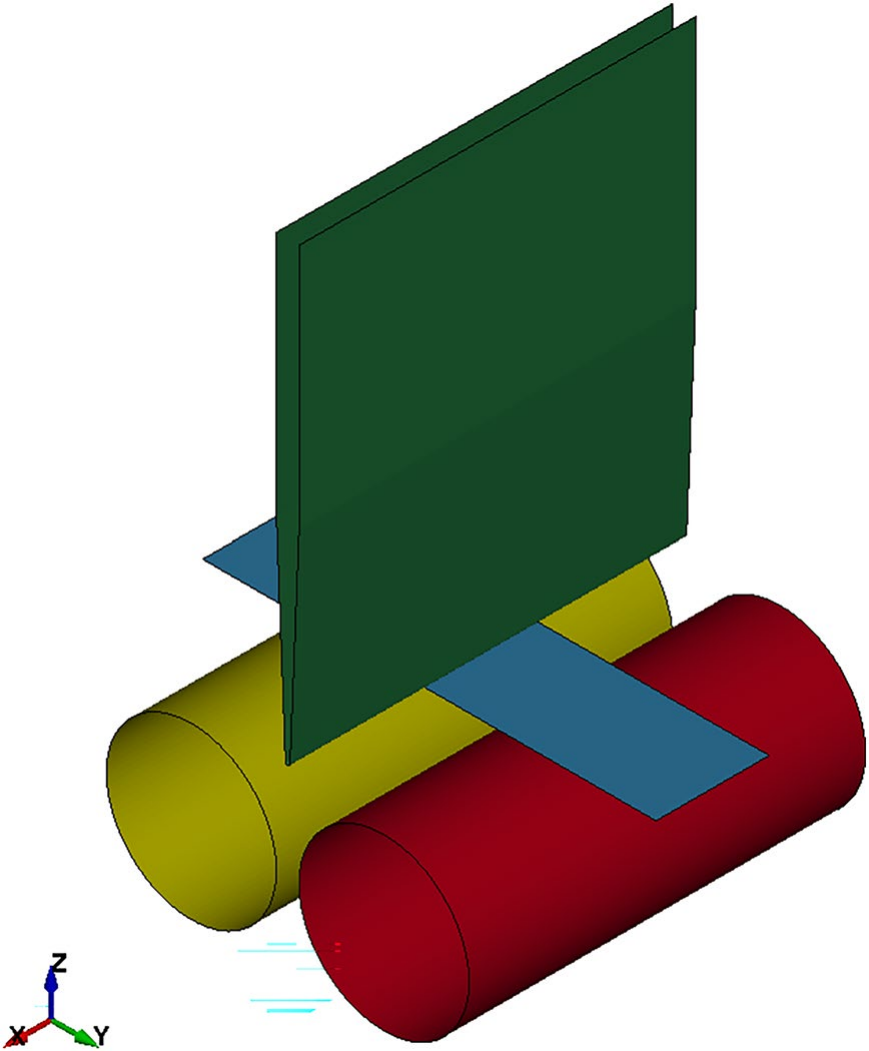
FE model of three-point-bending test used for validation

The experimental set-up for FE was designed with the CAD Software Autodesk Inventor, modeled with shell elements in Altair Hypermesh, and is shown in Fig. 3.

In the finite element analysis conducted with LS-DYNA R11, the material model *MAT_036 was used, as it is capable of handling deformation dependent elastic moduli as an tabular input. This material model is also able to implement several hardening models, can consider anisotropy via the usage of Lankford parameters and has a flow limit diagram failure option. Consequently it is used in industrial applications. In our case, only isotropic hardening via implementation of the measured flow curves was considered.

After forming, a springback analysis of the bent stripes was performed using the same material cards and compared to the experimentally determined angles. 

## Results and discussion

### Initial Young’s modulus determination

The determined initial elastic moduli are given in Table 3. Therein, differences in the determined average values and the repeatability (standard deviation) of each determination method are given.

**Table 3:** Initial Young’s modulus of the investigated light metal sheets

Method	EN AW-6082-T6 [GPa]	AZ31B [GPa]	Ti–6Al–4V [GPa]
LUS-res	70.43±0.08	44.42±0.18	112.83±0.06
US-tof	69.36±0.15	43.10±0.37	114.16±0.71
EN ISO 6892-1	71.23±0.55	45.15±0.38	120.16±0.84
Suttner-Merklein	72.00±0.44	45.17±0.40	121.50±0.46
3-point-bending	71.85±0.23	41.52±0.44	123.53±1.77

The three-point-bending experiments show a good repeatability for EN AW-6082-T6 and a suboptimal repeatability for Ti–6Al–4V. At the same time, the three-point-bending results can show either a significantly increased or reduced Young’s modulus compared to the other test methods. In particular the measured 41.52 GPa for AZ31B are 3.6 % to 8.1 % lower than the values measured via tensile tests and ultrasonic measurements. It is suspected that the used experimental set up is responsible for this, as the determined Young’s modulus is influenced by the experimental conditions, e.g. the punch radius [50] and the span length [51]. This explanation is supported by inter-laboratory tests, which revealed that the results of bending tests can vary greatly between different laboratories [21]. The reasons given for this are based on the test setup, the execution of the test and the practical experience of the laboratories conducting the experiments.

When the Suttner-Merklein method [47] is used, the determined Young’s modulus is slightly increased compared to the standardized method (EN ISO 6892-1). This deviation is partly associated with the usage of true stress and true strain values instead of engineering values for the calculation of the Young’s modulus. This changes the slope of the true stress/true strain curve slightly. The slope increases continuously in the elastic regime, while the slope of the engineering values is constant for ideal elastic behavior. Nevertheless, both determination methods show a good repeatability.

The applied ultrasonic measurement techniques, both categorized as dynamic methods for identifying the linear elastic modulus, show a tendency of a higher repeatability for the determination of the Young’s modulus compared to bending and/or tensile tests, which are generally categorized as static determination methods. Especially the LUS-res measurements show a very high repeatability, while the repeatability of the manually conducted Young’s modulus determination based on US-tof measurements is only high for the aluminum alloy. This may be due to coupling difficulties during the manual handling of the ultrasonic probes and lower thickness measurement accuracy.

However, the values determined via the conducted ultrasonic measurements are surprisingly lower than the tensile test based values, which is especially the case for the Ti–6Al–4V results. Based on the general literature, the opposite should be the case. An explanation for such an unusual behavior is given by Garlea et al. [52], who also reported this behavior for their experiments with the magnesium alloy AZ31B at elevated temperatures. They attributed the observed differences to the effect of anelastic mechanisms, such as grain boundary sliding (as the main mechanism) and creep. However, creep effects are not a sufficient explanation in our case, especially for titanium. It seams more likely that anisotropic material behavior may have played a role in this investigation. Depending on the orientation of the ultrasonic probes to the rolling direction, different transversal sound velocities were measured, e.g. for titanium 3177.6±10.6 m/s and 3062.4±12.7 m/s. Consequently, the unexpected low values of the ultrasonic based determination methods might be cased or affected by microstructural effects and uncertainties in the thickness and/or the density measurement, which affect the ultrasonic-based methods more than the determination of the Young’s modulus via tensile tests.

### Measurements of elastic properties during plastic deformation

**Fig. 4:**
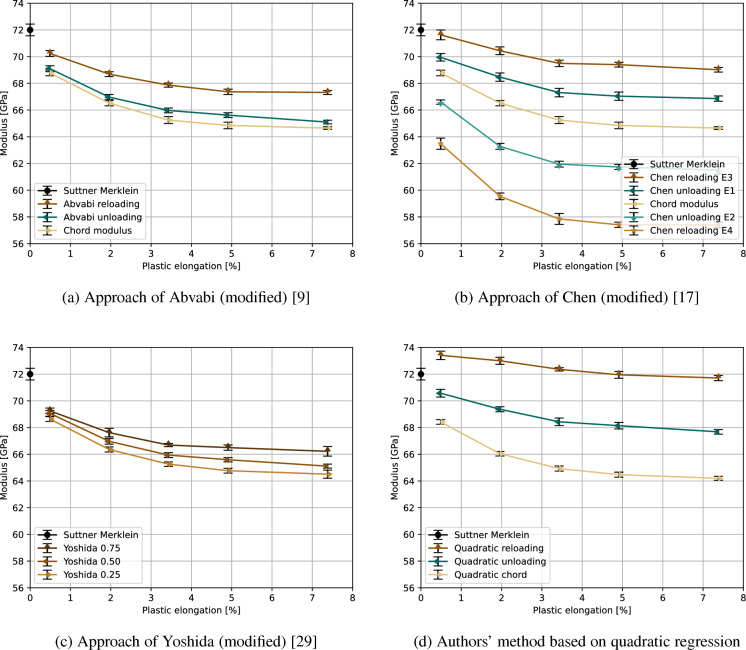
Comparison of the different methods to determine the strain-dependent elastic modulus of EN AW-6082

**Fig. 5:**
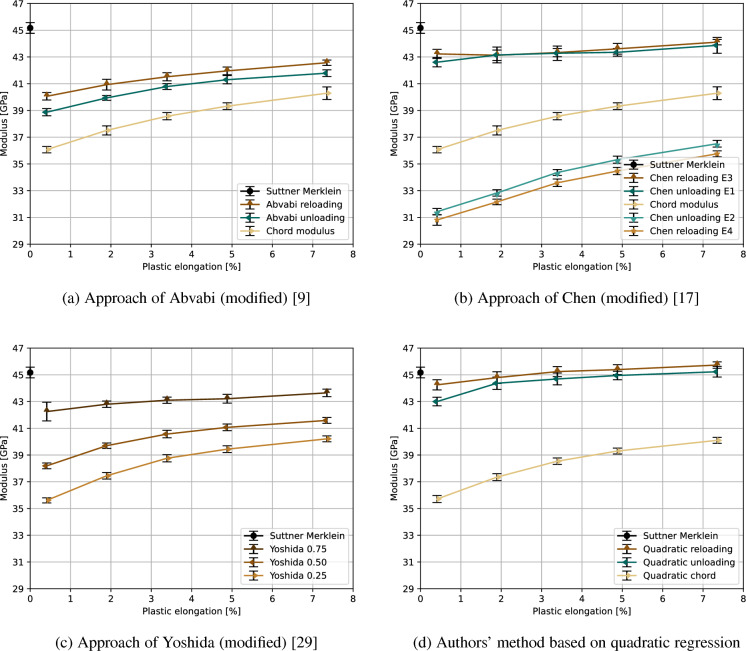
Comparison of the different methods to determine the strain-dependent elastic modulus of AZ31B

**Fig. 6:**
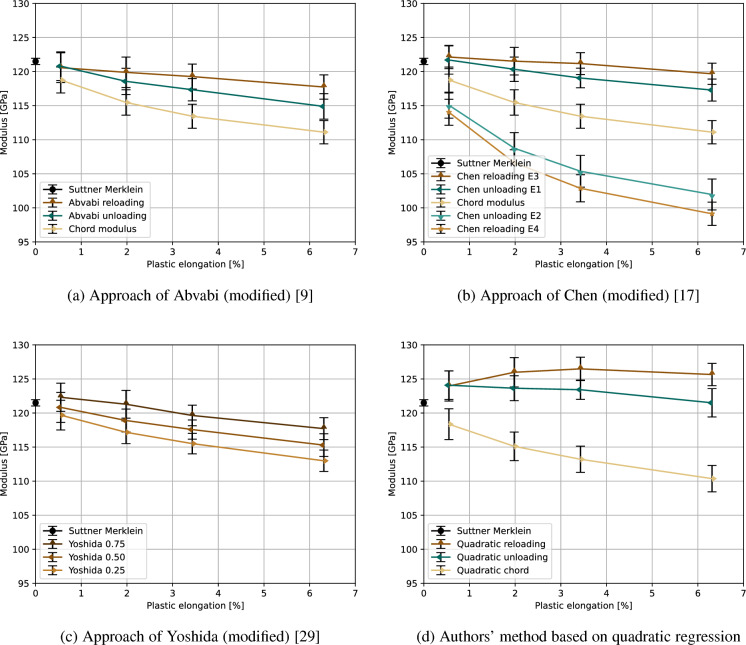
Comparison of the different methods used to determine the strain-dependent elastic modulus of the titanium alloy Ti–6Al–4V

The continuous loading-unloading-loading experiments reveal significant changes of the elastic moduli, see Figs. 4, 5, and 6 (error bars represent standard deviation). In general, the tendency frequently described in the literature of a decreasing modulus of elasticity caused by plastic deformation can be confirmed. However, significant differences between the investigated light metal alloys and the applied determination methods were found and are presented for each alloy separately.

The aluminum alloy EN AW-6082 is a good example for the elastic degradation with increasing plastic strain (Fig. 4). The elastic modulus decreases from its initial value of 72.0 GPa or 71.2 GPa (Suttner-Merklein or EN ISO 6892-1, respectively, see Table 3) to 57.4–69.0 GPa (depending on the tensile test-based determination method) due to plastic deformation. The large discrepancy in the determined elastic moduli depending on the method used is due to calculation of the moduli in different regions of the stress/strain curve. As the differences in the local slopes or the stress/strain curves can be quite high. Thereby, the slope of the hysteresis loop center line, which directly connects the turning point and the intersection, is quite low and therefore results in a low chord modulus. A comparison with other calculated moduli revealed that only the moduli determined at the unloading part of the curve just before turning point of the hysteresis (Chen unloading E2) and at the top of the reloading curve (Chen reloading E4) are lower than the chord modulus. All evaluation protocols applied for the investigated aluminum alloy also revealed a decrease in the reduction of the elastic moduli with an increasing plastic strain, which finally leads to saturation level. The value of this saturation level depends strictly on the evaluation method or, in other words, on the region of the slope of the stress/strain curve that is used for the calculation of the elastic moduli. It is worth noting that the authors’ method for determining the chord modulus results in a slightly lower standard deviation than the commonly used method (cf. error bars).

AZ31B shows drastically different behavior (Fig. 5). After a drop to 30.8–44.3 GPa (method-dependent), elastic modulus values increase to 35.8–45.7 GPa when a plastic strain of 7.35 % is reached during the tensile deformation. For the determination methods based on the literature, the values obtained at higher strains almost reach the initial Young’s modulus as determined via tensile test (approximately 45.2 GPa, see Table 3). Additionally, the authors’ evaluation methodology can even result in higher moduli of up to 45.7 GPa. However, the chord modulus shows a similar initial drop, then slightly increases to reach a value of 40.1 GPa at an elongation of approximately 7.5 %. Again, the authors’ method for determining the chord modulus exhibits a lower standard deviation than the commonly used method (cf. error bars).

This reveals no fundamental differences between the methods, as all approaches show an increase of the elastic moduli after an initial drop. Based on the findings of Iftikhar et al. [25] and Nguyen et al. [38] for magnesium alloys containing approximately 3 % Al and 1 % Zn, the elastic moduli should decrease with increasing plastic deformation until a saturation level is reached. Nevertheless, other authors describe an increase of the elastic modulus due to plastic deformation after an initial drop [53–55]. An explanation for this unusual behavior might be the change of the dominant deformation mechanism. According to Yan et al. [53], it could be possible that the change of the deformation mechanism from slipping to twinning leads to instant stress relaxation among the grains, which may affect the atom binding force. Based deformation experiments with ZE10 sheets and crystal plasticity modeling Tang et al. [54] concluded that the inelastic behavior and the chord modulus can be explained by basal slip, twinning and de-twinning during the unloading process. Their research also revealed a strong dependency on the sampling direction and the loading path, due to a weak basal-type texture with the c-axis tilted towards the transverse direction, which also explains different twinning activities and consequently higher chord moduli in rolling direction.

The titanium alloy Ti–6Al–4V, which has a duplex microstructure, responds to the applied tensile deformation as described in the literature (Fig. 6). Depending on the tensile test-based determination method, the elastic modulus decreases in most cases from its initial value of 121.5 GPa or 120.2 GPa (Suttner-Merklein or EN ISO 6892-1, respectively, see Table 3) to 99.1–119.7 GPa (method-dependent) due to plastic deformation. In contrast to this, the reloading modulus of the authors’ evaluation protocol initially shows an increase due to plastic deformation up to 126.5 GPa, followed by a slight decrease upon further deformation. Additionally, also the other investigated measurement methods show a slight increase of some elastic moduli at lower strain of approximately 0.5 % before these moduli starts to decrease, see Fig. 6.

Based on these results, it can be concluded that the recorded change of the elastic moduli depends on the material and the measurement method, whereby it is still unclear which measurement method is most suitable for the implementation in FE simulations. The next section seeks to elucidate this question.

### Validation by three-point-bending tests and FE simulations

**Fig. 7:**
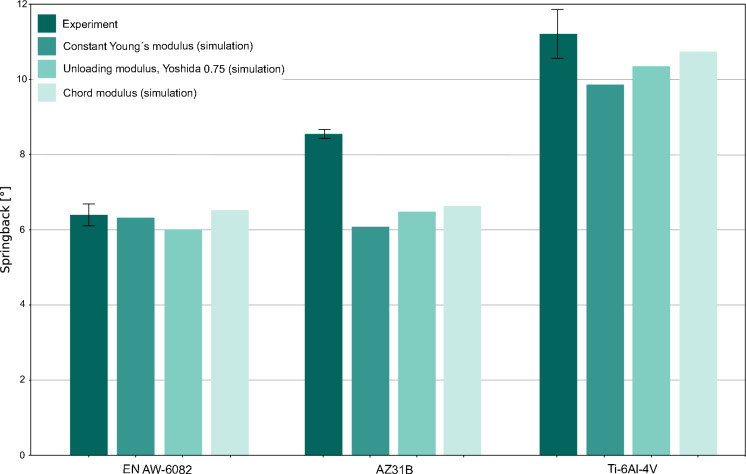
Comparison of numerical springback prediction with three-point-bending experiments of light metal alloys

A practice-oriented validation requires the comparison of modeling approaches that not only takes into account the reduction of the modulus of elasticity, but also the used hardening law. Therefore, validating the implemented material model, which only considers isotropic hardening, for the three-point-bending of the investigated light alloys was necessary. In this first step, the chord modulus and Yoshida et al. [29] unloading modulus were used, as these have been already employed successfully for springback prediction in the literature [5, 16]. Furthermore, simulations with a constant Young’s modulus were conducted for comparison. In Fig. 7, the springback after three-point-bending of the three alloys is shown together with these FE results (error bars represent standard deviation).

It can be seen that the implemented hardening model seems to be capable for the intended purpose, as the results of the FE simulation are in most cases comparable with the bending experiments. Especially for EN AW-6082, the difference between the springback prediction and the validation experiment is quite small: The implementation of the Yoshida et al. [29] 0.75 modulus yields a deviation of 6.1 % between experiment and simulation, while usage of the chord modulus and the constant Young’s modulus lead to deviations of 2.0 % and 1.2 % respectively.

However, the agreement between simulation and experiment for the magnesium and titanium alloy simulations is not as good as for the aluminum alloy. For Ti–6Al–4V, deviations range from 4.2–12 %, whereby the use of a constant Young’s modulus resulted in the largest deviation.

The results of the AZ31B bending simulation show a deviation of up to 28.9 %. Similar to the titanium results, neglecting the weakening of the elastic moduli leads to the lowest agreement between simulation and experiment.

The incorporation of the changing elastic modulus caused by plastic deformation via the usage of the chord modulus leads to an improvement of simulation accuracy by approximately 8 % for titanium and 6 % for magnesium. While the accuracy may then be considered acceptable for Ti–6Al–4V, the deviation is still unsatisfactory for AZ31B.

**Fig. 8:**
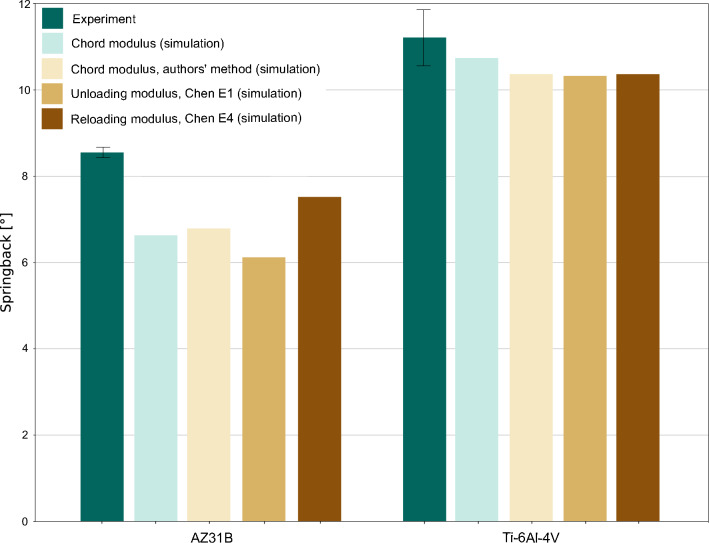
Comparison of springback prediction with experimental results of AZ31B and Ti–6Al–4V three-point-bending tests

The influence of other elastic modulus determination methods is examined in more detail in Fig. 8 (error bars represent standard deviation). Therein, results of using the determination technique according to Chen et al. [17] and the authors method , which uses quadratic regression to determine the ends of the corresponding chord, are shown. The results obtained by utilizing the commonly used chord modulus are given again for convenient comparison. The implementation of the Chen unloading modulus E1, the Chen reloading modulus E4, a determination method which tends to generate quite low elastic moduli, and the authors chord modulus, which deviates slightly from the commonly used chord modulus, covers a wider range of the determined elastic values (cf. Chapter 4.2).

For the magnesium alloy, a strong influence of the elasticity parameter used can be clearly seen (Fig. 8). The use of high elastic moduli (e.g., unloading modulus, Chen E1) leads to a high deviation, while the usage of very low values (reloading modulus, Chen E1) is able to increase the accuracy of the springback prediction up to a deviation of 12 %, which is, in this case, one degree in bending angle.

In case of Ti–6Al–4V, such a clear trend is not visible (Fig. 8). Nevertheless, the consideration of the elastic moduli degradation by employing the chord modulus is still beneficial compared to the usage of a constant Young’s modulus.

Our results indicate that the result of the elastic modulus determination strongly depend on the material and determination technique. The trend described in the literature of an improved springback prediction when using changing (strain-dependent) elastic moduli [5, 7, 8] was confirmed for most of the investigated light metals. However, for EN AW-6082, the implementation of a constant Young’s modulus also generated very accurate springback predictions.

Chang et al. [8] also reported that a constant Young’s modulus showed a better agreement between simulation and experiment then variable elastic moduli for a bending angle of 60$^\circ$. Nevertheless, their results also showed that the consideration of the elastic moduli degradation is beneficial for bending angles of 90$^\circ$ and 120$^\circ$. Furthermore, geometrical factors such as bending angle and punch radius can influence the difference between simulated and tested springback [8].

The results suggest that the chord modulus determined from true stress/strain data is generally a good choice to describe the phenomenon of elastic modulus reduction caused by plastic deformation properly. At the same time, it was demonstrated that the utilization of an alternative determination method, which results in a greater reduction of the modulus of elasticity, can be occasionally more advantageous.

Our work has certain limitations, notably the omission of pertinent material effects, such as the Bauschinger effect or time-dependent springback. Furthermore, the results from this research should not be directly transferred to complex multi-directional bending operations, deep drawing or rotary draw bending. The consideration of changing stress states is necessary to achieve a high accuracy in FE simulations of these processes [16, 26, 56].

## Conclusions

Based on the results of the comparative measurement study, the determination of the initial Young’s modulus via non-destructive ultrasonic testing techniques is advisable. Especially the laser ultrasonic technique exhibits superior accuracy in determining the Young’s modulus of light metal sheets. At higher sample thicknesses, time-of-flight based measurements can be an cost-effective alternative.

Regarding the determination of changes of the moduli of elasticity caused by plastic deformation, the following conclusions can be made:

- Aluminum and titanium alloys show strong elastic degradation with increasing plastic strain.
- Magnesium alloy AZ31B showed an increase of the determined elastic moduli after an initial drop.
- Depending on the used tensile test-based determination method, the calculated elastic moduli values can differ significantly. The repeatability of the chord modulus determination could be slightly improved by implementing the authors’ method for the aluminum and magnesium alloys.
- The implementation of a strain-dependent modulus of elasticity can increase the accuracy of springback simulations. However, for EN AW-6082, no benefit was found.
- The implementation of the commonly utilized chord modulus is the first choice to consider the effect of elastic degradation.
- However, depending on the material (here: AZ31B), the bending angle, and the boundary conditions of the deformation process, the usage of alternative elastic moduli can be beneficial.

Further research should combine more complex material models with different elastic moduli determination techniques in order to increase our understanding of the springback behavior of light metals.

## Data Availability

The raw and processed data required to reproduce these findings are available upon reasonable request.
